# Synthesis and Preliminary Evaluation of *N*-Oxide Derivatives for the Prevention of Atherothrombotic Events

**DOI:** 10.3390/molecules201018185

**Published:** 2015-10-07

**Authors:** Leandro Augusto Rosseto, Maria Elisa Lopes Pires, Aylime Castanho Bolognesi Melchior, Priscila Longhin Bosquesi, Aline Renata Pavan, Sisi Marcondes, Man Chin Chung, Jean Leandro dos Santos

**Affiliations:** 1 Faculdade de Ciências Farmacêuticas, Universidade Estadual Paulista, UNESP, Rodovia Araraquara Jaú Km 01, 14801-902, Araraquara, SP, Brazil; E-Mails: leandroaugusto.rosseto@gmail.com (L.A.R.); mimi.castanho@gmail.com (A.C.B.M.); bosquesi@fcfar.unesp.br (P.L.B.); alinerenatapavan2004@yahoo.com.br (A.R.P.); chungmc@fcfar.unesp.br (M.C.C.); 2 Departmento de Farmacologia, Faculdade de Ciências Médicas, Universidade Estadual de Campinas, UNICAMP, Rua Tessália Vieira de Camargon.126, 13083-887, Campinas, SP, Brazil; E-Mails: mariaelisalopes@yahoo.com.br (M.E.L.P.); sisi@fcm.unicamp.br (S.M.)

**Keywords:** *N*-oxide, atherothrombosis, antiplatelet activity, bleeding time, furoxan, benzofuroxan, genotoxicity

## Abstract

Thrombosis is the main outcome of many cardiovascular diseases. Current treatments to prevent thrombotic events involve the long-term use of antiplatelet drugs. However, this therapy has several limitations, thereby justifying the development of new drugs. A series of *N*-oxide derivatives (furoxan and benzofuroxan) were synthesized and characterized as potential antiplatelet/antithrombotic compounds. All compounds (**3a**,**b**, **4a**,**b**, **8a**,**b**, **9a**,**b**, **13a**,**b** and **14a**,**b**) inhibited platelet aggregation induced by adenosine-5-diphosphate, collagen, and arachidonic acid. All compounds protected mice from pulmonary thromboembolism induced by a mixture of collagen and epinephrine; however, benzofuroxan derivatives (**13a**,**b** and **14a**,**b**) were the most active compounds, reducing thromboembolic events by up to 80%. *N*-oxide derivative **14a** did not induce genotoxicity *in vivo*. In conclusion, **14a** has emerged as a new antiplatelet/antithrombotic prototype useful for the prevention of atherothrombotic events.

## 1. Introduction

Cardiovascular diseases (CVD) are the main cause of death worldwide. In 2012, approximately 17.5 million people died from CVD, which represents 31% of all deaths worldwide in that year. Estimative data suggest that deaths by CVD could reach 25 million in 2030 [1], with thrombosis being one of the primary outcomes [2].

Currently, antiplatelet drugs (APDs) represent one of the major classes of antithrombotic drugs. In 2013, APD sales reached 9.5 billion dollars, accounting for 40.4% of all antithrombotic drugs [3]. APDs target key pathways on the platelet surface, namely adenosine-5-diphosphate (ADP)-mediated signaling, thromboxane A2 synthesis, and integrin αIIbβ3. In long-term therapy, APDs prevent clot formation and vaso-occlusion [4]. Despite the clinical advantages of APD therapy, recurrent vascular events still occur in 10%–20% of all patients under treatment [5]. In addition, 5%–40% of coronary patients treated with APDs do not show a protective response [6]. 

Current antiplatelet/antithrombotic therapies have several limitations, which include blocking a single pathway of platelet aggregation, increase in bleeding time, weak inhibition of platelet aggregation, high variability in the patients’ responses, and slow onset of action [7]. Recently, the term “resistance” emerged in the literature to describe the limited efficacy of drugs, such as acetylsalicylic acid (ASA) and clopidogrel. The percentage of drug-resistant individuals ranged from 4%–30% among the patients [8]. This resistance is related to factors such as increased activity of alternative pathways in platelets, drug-drug interactions, pharmacogenetic factors, and poor adherence to the therapy [8,9]. 

**Figure 1:**
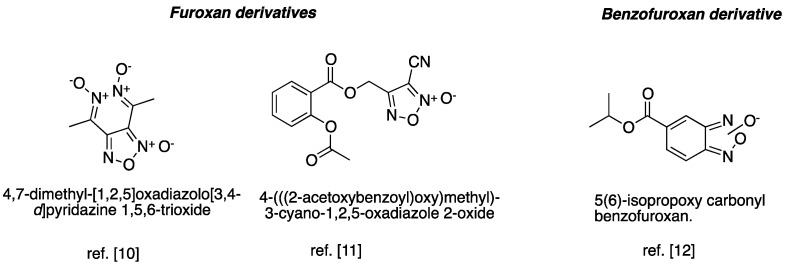
Chemical structures of furoxan and benzofuroxan derivatives with antiplatelet activity [10,11,12].

Therefore, the discovery of safe and effective compounds to prevent thromboembolic events is a milestone that still needs to be overcome in the current therapy. *N*-oxide derivatives (e.g., furoxan and benzofuroxan) have been described as promising prototypes to design new compounds to treat cardiovascular disorders due to their antiplatelet activity [10,11,12] (Figure 1). Therefore, in a continuing effort to develop safe and effective antiplatelet and antithrombotic drug candidates, we report on the synthesis and pharmacological evaluation of new *N*-oxide derivatives that may prevent atherothrombotic events. 

## 2. Results and Discussion

### 2.1. Chemistry

**Scheme 1:**
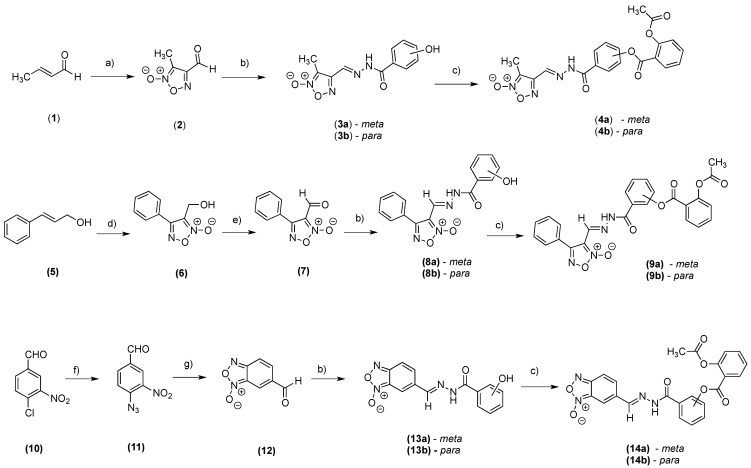
Synthesis of furoxan (**3a**,**b**, **4a**,**b**, **8a**,**b** and **9a**,**b**) and benzofuroxan derivatives (**13a**,**b** and **14a**,**b**).

The synthetic routes for preparing the intermediates (**3a**,**b**, **8a**,**b**, **13a**,**b**) and final compounds (**4a**,**b**, **9a**,**b**, **14a**,**b**) are summarized in Scheme 1. The furoxan (**2** and **6**) and benzofuroxan (**12**) subunits were prepared according to previously described procedures [13,14]. The intermediates (**3a**,**b**, **8a**,**b**, **13a**,**b**) were prepared by condensation between aldehyde groups and various hydroxybenzhydrazides. These intermediates were obtained with variable yields of 75%–95% and the ^1^H-nuclear magnetic resonance (NMR) spectra of all intermediates showed one signal corresponding to the ylidenic hydrogen of the *E*-diastereomers [13,15]. 

The final compounds (**4a**,**b**, **9a**,**b**, **14a**,**b**) were prepared in variable yields (21.6%–39.1%) through esterification between the carboxylic acid present in ASA and the hydroxyl group present in the intermediates (**3a**,**b**, **8a**,**b**, **13a**,**b**) using *N*,*N*′-dicyclohexylcarbodiimide as a coupling agent. All chemical structures were established by elemental analysis, infrared (IR) spectroscopy, and ^1^H- and ^13^C-NMR. The purities of all compounds, as determined by high-performance liquid chromatography, were >98.5%. 

**a**) NaNO_2_, CH_3_COOH, 0 °C, 24 h, 38%; **b**) hydroxybenzhydrazide derivative, ethanol, acid catalysis, 25 °C, 15 h, 38%–68%; **c**) DCC, dichloromethane, 25 °C, 15 h, 21.6%–39.1%; **d**) NaNO_2_, CH_3_COOH, 0 °C, 24 h, 90%; **e**) Pyridinium chlorochromate, CH_2_Cl_2_, 25 °C, 24 h, 45%; **f**) NaN_3_, DMSO, 75 °C, 1 h, 99%; **g**) toluene, 120 °C, 1.5 h, 78%.

### 2.2. Pharmacological Evaluation

#### 2.2.1. Antiplatelet Activity and Nitric Oxide Release 

*In vitro* antiplatelet activity was evaluated using rat platelet-rich plasma in the presence of ADP (10 μM), arachidonic acid (100 μM) and collagen (5 μg/mL) [15,16]. Table 1 shows that all compounds (**3a**,**b**, **4a**,**b**, **8a**,**b**, **9a**,**b**, **13a**,**b** and **14a**,**b**) at 150 μM inhibit platelet aggregation induced by all those agonists used. Antiplatelet activity induced by ADP ranged from 9.2% to 75.6% and activity induced by arachidonic acid ranged from 31.6% to 87.5%. For collagen, the antiplatelet inhibition ranged from 8.8% to 85.3%. All synthesized compounds demonstrated antiplatelet activity against ADP-induced platelet aggregation, whereas the parental drug (ASA) did not inhibit this pathway. 

**Table 1:** Antiplatelet activity (%) and NO release data of furoxan (**3a**,**b**, **4a**,**b**, **8a**,**b** and **9a**,**b**) and benzofuroxan derivatives (**13a**,**b** and **14a**,**b**).

Compounds	Platelet Inhibition (%) ^a^			NO Release Data
	ADP (10 μM)	Arachidonic Acid (100 μM)	Collagen (5 μg/mL)	%NO_2_^−^ (mol/mol) ^b^, 50 × 10^−4^ M l-Cys
Control ^c^	0	0	0	0
DNS	N.D. ^d^	N.D. ^d^	N.D. ^d^	10.5 ± 0.7
ASA	0	72.2 ± 3.7 *	77.1 ± 3.7 *	0
**3a**	10.1 ± 3.3 *	52.6 ± 4.1 *	17.5 ± 2.7 *	2.0 ± 0.7
**3b**	16.8 ± 2.9 *	47.8 ± 3.7 *	10.5 ± 3.3 *	1.9 ± 1.1
**4a**	65.6 ± 2.4 ^†,^*	70.1 ± 2.9 *	37.8 ± 4.1 *	2.2 ± 0.4
**4b**	75.6 ± 1.8 ^†,^*	87.5 ± 2.6 ^†,^*	34.5 ± 3.5 *	1.8 ± 1.7
**8a**	12.4 ± 3.7 ^†,^*	55.1 ± 4.3 *	21.8 ± 5.1 *	7.6 ± 1.2
**8b**	9.2 ± 2.8 ^†,^*	31.6 ± 3.9 *	8.8 ± 3.6 *	7.5 ± 0.9
**9a**	53.4 ± 2.1 ^†,^*	73.4 ± 3.3 *	43.5 ± 4.7 *	7.0 ± 0.8
**9b**	68.8 ± 1.3 ^†,^*	82.7 ± 2.1 ^†,^*	47.2 ± 2.9 *	7.9 ± 1.3
**13a**	47.1 ± 2.5 *	52.3 ± 3.8 *	45.1 ± 3.8 *	0
**13b**	44.2 ± 5.1 ^†,^*	57.8 ± 4.9 *	43.0 ± 3.1 *	0
**14a**	74.9 ± 2.2 ^†,^*	76.9 ± 3.1 *	85.3 ± 3.7 *	0
**14b**	70.3 ± 1.7 ^†,^*	79.1 ± 1.9 ^†,^*	79.8 ± 3.4 *	0

^a^ Results are expressed as the mean for *n* = 3 independent experiments carried out in triplicate. All compounds and ASA were evaluated at 150 μM; ^b^ All values are the mean ± SEM. Determined by Griess reaction, after incubation for 1 h at 37 °C in pH 7.4 buffered water, in the presence of 1:50 molar excess of l-cysteine; ^c^ Vehicle DMSO (0.1% *v*/*v*) was used as a control; ^d^ N.D. not determined; * *p* < 0.01 versus the control group and ^†^ *p* < 0.01 versus ASA (ANOVA followed by Tukey test).

Compounds **4b**, **14a** and **14b** inhibited more than 70% of platelet aggregation induced by ADP. Interestingly, benzofuroxan derivatives (**14a**,**b**) showed antiplatelet activity superior to ASA in experiments using arachidonic acid and collagen as agonists. It was observed that benzofuroxan derivatives (**14a**,**b**) were more active as antiplatelet compounds than furoxan derivatives (**4a**,**b**, **9a**,**b**). Furthermore, it was verified that those compounds containing the 2-acetoxybenzoyl subunit (**4a**,**b**, **9a**,**b**, **14a**,**b**) had antiplatelet activity superior to their respective intermediates (**3a**,**b**, **8a**,**b**, **13a**,**b**) in the presence of all three agonists. 

Nitrite quantification was used as an indirect method to evaluate the NO donor ability of all compounds (**3a**,**b**, **4a**,**b**, **8a**,**b**, **9a**,**b**, **13a**,**b** and **14a**,**b**) *in vitro* [17]. The results of thiol-induced NO generation after incubation with a high concentration of l-cysteine (50 × 10^−4^ M) are shown in Table 1. Compounds induced nitrite formation at levels of 0%–7.9%. Isosorbide dinitrate (DNS), used as the control, induced nitrite formation at a level of 10.5%. Furoxan derivatives **3a**,**b** and **4a**,**b** demonstrated a similar ability to generate nitrite in medium. Such results were also observed for furoxan **8a**,**b** and **9a**,**b**. Benzofuroxan derivatives (**13a**,**b** and **14a**,**b**) did not generate nitrite in the medium. In addition, it was characterized that the presence of the 2-acetoxybenzoyl subunit did not contribute to the release of NO in furoxan derivatives, suggesting that the antiplatelet effects of these compounds (**3a**,**b**, **4a**,**b**, **8a**,**b**, **9a**,**b**, **13a**,**b** and **14a**,**b**) could be independent of the NO action. 

#### 2.2.2. *In Vivo* Antithrombotic Activity 

The antithrombotic effects of all compounds (**3a**,**b**, **4a**,**b**, **8a**,**b**, **9a**,**b**, **13a**,**b** and **14a**,**b**) were evaluated *in vivo* using a mouse pulmonary thromboembolism model [18,19]. All compounds, after oral administration in a single dose, protected mice against thromboembolic events (Table 2). 

**Table 2:** *In vivo* antithrombotic effect of compounds (**3a**,**b**, **4a**,**b**, **8a**,**b**, **9a**,**b**, **13a**,**b** and **14a**,**b**) and acetylsalicylic acid (ASA) using an acute pulmonary thromboembolism model induced by a collagen-epinephrine mixture.

Compounds	Paralyzed ^a^ or Died Animals/Total	% Protection
Control	9/10	10
ASA	7/10	30 *
**3a**	6/10	40 *
**3b**	7/10	30 *
**4a**	6/10	40 *
**4b**	5/10	50 *^,^†
**8a**	6/10	40 *
**8b**	6/10	40 *
**9a**	7/10	30 *
**9b**	5/10	50 *^,†^
**13a**	3/10	70 *^,†^
**13b**	3/10	70 *^,†^
**14a**	2/10	80 *^,†^
**14b**	3/10	70 *^,†^

The test compounds were orally administered at a dose of 100 µmol/10 g body weight (0.1 mL/10 g body weight). The χ^2^ test was used to compare the survival rate between the control and treated groups. * *p* < 0.05 compared with the control group and ^†^ *p* < 0.05 compared with ASA. ^a^ The loss of the righting reflex was considered to indicate paralysis.

The antithrombotic activities of **4b**, **9b**, **13a**,**b**, and **14a**,**b** were superior to that of ASA, used as a control. The benzofuroxan derivatives (**13a**,**b** and **14a**,**b**) were the most effective compounds, providing a survival of up to 80%. It was observed that compounds containing the 2-acetoxybenzoyl subunit (**4b**, **9b**, and **14a**) were more active than their intermediates (**3b**, **8b**, and **13a**).

#### 2.2.3. *In Silico* Studies

**Figure 2:**
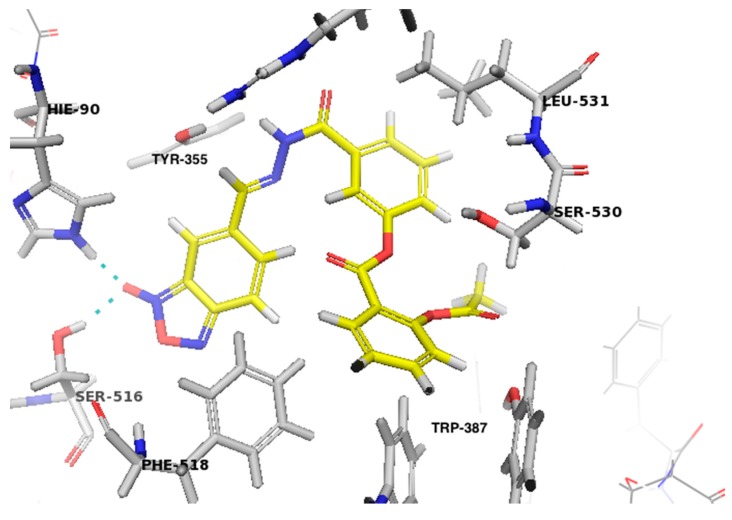
Docking study of **14a** (yellow colored carbon atoms) in the active site of COX-1 (3KK6, residues labeled in gray for carbon). The hydrogen bond between *N*-oxide hydrogen and His90 and Ser516 (3.1 Å) residues is depicted by the dashed cyan line. The figure was generated using Pymol software (version 1.7.6).

Benzofuroxan derivatives have been described as inhibitors of cyclooxygenase (COX) enzymes, mainly for the isoform COX-1 [20]. Therefore, the ability of the benzofuroxan derivative (**14a**) to interact with COX-1 (PDB ID: 3KK6) and its mode of binding were investigated through docking studies using software Maestro v9.1 (Schrödinger^®^, New York, NY, USA). Figure 2 shows the main interactions of **14a** with amino acid residues present in the active site of COX-1. The orientation of benzofuroxan **14a** showed that the *N*-oxide subunit interacts through hydrogen bonding with His90 and Ser516 (3.1 Å). The amidic hydrogen of the *N*-acylhydrazone subunit interacts with residue Tyr355 (3.3 Å). Furthermore, several hydrophobic interactions [21] were characterized between **14a** and hydrophobic residues in the active site such as Leu531, Trp387, and Phe518 (less than 4.5 Å). This result could explain, in part, the antiplatelet effect observed for this compound; however, additional mechanisms need to be further investigated.

The *in silico* physico-chemistry and pharmacokinetic properties of **14a** were determined using the pkCSM database [22]. Table 3 shows that **14a** does not violate Lipinski’s rule of five and has an adequate pharmacokinetic profile for oral administration [23]. 

**Table 3:** Physico-chemistry properties and ADMET profile of **14a** using pkCSM database [22].

Predicted properties	14a
Physico-chemistry properties	Molecular weight: 460.1 H donors: 1 H acceptors: 9 LogP: 2.36 Number of rotatable bonds: 9 Surface area: 191.27
Absorption	Water solubility: −4.71 log mol/L Caco2 permeability: −0.008 log Papp in 10^−6^ cm/s Intestinal absorption (humans): 86.54% absorbed
Distribution	VDss (humans): −0.869 log L/Kg
Metabolism	Substrate of CYP3A4
Excretion	Total clearance: 0.386 log mL/min/Kg
Toxicity	AMES toxicity: No hERG I inhibitor: No

#### 2.2.4. *In Vivo* Genotoxicity Studies

**Figure 3:**
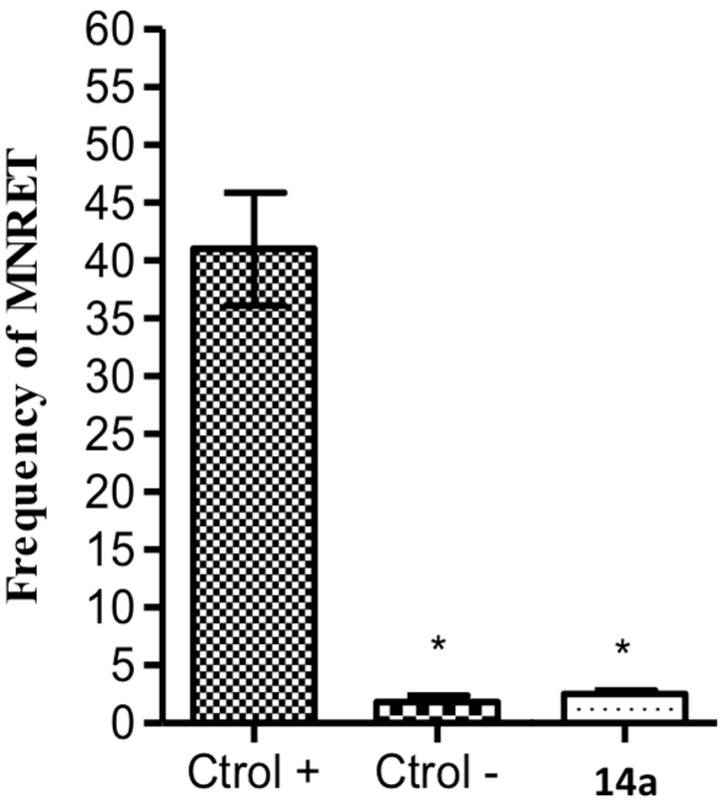
Mean ± SD frequency of MNRET in 1000 cells obtained from mice treated with cyclophosphamide (50 mg/kg, positive control), CMC/Tween (negative control), and compound **14a** (250 mg/kg). * *p* < 0.05 (compared with the positive control; ANOVA followed by Tukey test).

The genotoxicity induced by **14a** was evaluated using the micronucleus test [24]. Compound **14a** was selected for the genotoxicity studies because *N*-oxide compounds, especially those containing the benzofuroxan subunit, have been associated with mutagenicity and genotoxicity [25,26]. Cyclophosphamide, used as positive control, induced a mean of 41 ± 5 micronucleated reticulocytes (MNRET) (Figure 3), whereas **14a** was not able to cause genotoxicity (MNRET < 4). For **14a**, MNRET values were similar to those present in the vehicle (CMC/Tween; negative control). 

## 3. Experimental Section 

### 3.1. General

The progress of all reactions was monitored by TLC, which was performed on 2.0 × 6.0 cm^2^ aluminum sheets pre coated with silica gel 60 (HF-254, Merck, Kenilworth, NJ, USA) to a thickness of 0.25 mm. The developed chromatograms were viewed under UV light (254 nm and 365 nm). Merck silica gel (70–230 mesh) was used for preparative column chromatography. Melting points were measured with an electrothermal melting-point apparatus (SMP3, Bibby Stuart Scientific, Stone, Staffordshire, UK) in open capillary tubes. Infrared spectra (KBr disc) were produced on an FTIR-8300 Shimadzu (Kyoto, Japan). ^1^H-NMR and ^13^C-NMR spectra were scanned on a Bruker DRX-400 (300 MHz) NMR spectrometer (Billerica, MA, USA) using DMSO-*d*_6_ as the solvent. The coupling constants are reported in hertz (Hz) and signal multiplicities are reported as singlet (s), doublet (d), doublet of doublet (dd), multiplet (m). Mass spectrometry of all compounds was obtained in positive mode using a model micrOTOF electrospray ionization-time of flight (ESI-TOF) (Bruker Daltonics, Billerica, MA, USA) spectrometer (conditions: injection at a flow rate of 300 L/h, capillary voltage of 4.5 kV, cone voltage of 120 V, desolvation temperature at 180 °C). Elemental analyses (C, H, and N) were performed on a Perkin Elmer model 240C analyzer (Waltham, MA, USA) and the data were within ±0.4% of the theoretical values. HPLC analysis was performed on a Shimadzu LC-10AD chromatograph equipped with a model SPD-10A UV-Vis detector (Shimadzu, Kyoto, Japan). The compounds were separated on a reversed phase C18 column (5 μm particle, 250 mm × 4.6 mm I.D.) Shimadzu Shim-pack CLC-ODS (M). HPLC-grade solvents (acetonitrile, methanol, and acetic acid) were used in the analyses and were bought from a local supplier. Reagents and solvents were purchased from commercial suppliers and used as received. The compounds aldehydes (**3a**), (**3b**), and (**4**) were prepared according methods previously described [13,14,27]. 

### 3.2. Chemistry

#### 3.2.1. General Procedures for the Preparation of Intermediates (**3a**,**b**, **8a**,**b** and **13a**,**b**) 

The corresponding aromatic aldehyde (**2**, **7**, or **12**) (2 mmol) was added to a solution of previously selected 3- or 4-hydroxybenzhydrazide derivatives (2 mmol) in 15 mL of absolute ethanol, in the presence of a catalytic amount of 37% hydrochloric acid (80 µL). The reaction was stirred for 15 h at room temperature and monitored by TLC. Afterwards, the solvent was partially concentrated at reduced pressure, and the resulting mixture was poured into cold water. The precipitate was collected by filtration, washed with cold ethanol, and dried under vacuum to give the intermediates containing the *N-*acyl hydrazone subunit (**3a**,**b**, **8a**,**b** and **13a**,**b**). If necessary, the compounds can be purified by column chromatography (flash silica, eluent: 70% ethyl acetate; 30% hexane). 

*3-((2-(3-Hydroxybenzoyl)hydrazono)methyl)-4-methyl-1,2,5-oxadiazole 2-oxide* (**3a**): Pale yellow solid. Yield: 47.2%, mp: 225–227 °C. IR νmax (cm^−1^; KBr pellets): 3414 (O-H), 1635 (C=O amide), 1616 (C=N), 1458 (C-H methyl), 1300 (N-O) and 800 (C-H aromatic). ^1^H-NMR (300 MHz, DMSO-*d*_6_) δ: 9.80 (1H; O-*H*), 8.46 (1H; s; C-*H*_imine_), 7.36 (1H; t; *J* = 7.9 Hz; C-*H*_aromatic_), 7.34 (1H; t; *J* = 7.9 Hz; C-*H*_aromatic_), 7.31 (1H; t; *J* = 7.9 Hz; C-*H*_aromatic_), 7.01 (1H; d; C-*H*_aromatic_), 7 (1H; N-*H*_amide_), 2.39 (3H; s; C-*H*_methyl_) ppm. ^13^C-NMR (75 MHz, DMSO-*d*_6_) δ: 137.7, 129.5, 119.2, 118.1, 114.7, 9.0 ppm. Analysis calculated for C_11_H_10_N_4_O_4_: C, 50.38; H, 3.84; N, 21.37. Found: C, 50.11; H, 3.82; N, 21.21. Mass calculated: *m*/*z* 262.07; Found Low-resolution mass spectrometry (LRMS): *m*/*z* 263.07 [M + H]^+^.

*3-((2-(4-Hydroxybenzoyl)hydrazono)methyl)-4-methyl-1,2,5-oxadiazole 2-oxide* (**3b**): Pale yellow solid. Yield: 48.35%, mp: 250–253 °C. IR νmax (cm^−1^; KBr pellets): 3316 (O-H), 2922 (C-H aromatic), 1617 (C=O amide), 1507 (N=O) and 1277 (C-H methyl). ^1^H-NMR (300 MHz, DMSO-*d*_6_) δ: 9.60 (1H; O-*H*), 8.49 (1H; s; C-*H*_imine_), 7.67 (2H; dd; *J* = 7.6 Hz; C-*H*_aromatic_), 6.72 (2H; dd; *J* = 7.6 Hz; C-*H*_aromatic_), 4.46 (1H; N-*H*_amide_), 2.08 (3H; s; C-*H*_methyl_) ppm. ^13^C-NMR (75 MHz, DMSO-*d*_6_) δ: 166, 160, 128, 124, 114, 71.6, 69.6, 26.5 ppm. Analysis calculated for C_11_H_10_N_4_O_4_: C, 50.38; H, 3.84; N, 21.37. Found: C, 50.3; H, 3.81; N, 21.5. Mass calculated: *m*/*z* 262.07; Found LRMS: *m*/*z* 263.07 [M + H]^+^.

*3-((2-(3-Hydroxybenzoyl)hydrazono)methyl)-4-phenyl-1,2,5-oxadiazole 2-oxide* (**8a**): White solid. Yield: 39.1%, mp: 236.1–237.5 °C. IR νmax (cm^−1^; KBr pellets): 3412 (O-H), 1653 (C=O amide), 1616 (C=N), 1400 (N=O), 1303 (N-O), 833 (C-H aromatic) and 744 (C-H aromatic). ^1^H-NMR (300 MHz, DMSO-*d*_6_) δ: 9.84 (1H; O-*H*), 8.37 (1H; s; C-*H*_imine_), 7.98 (2H; d; *J* = 8.1 Hz; C-*H*_aromatic_), 7.67 (1H; d; C-*H*_aromatic_), 7.61 (2H; d; *J* = 8.1 Hz; C-*H*_aromatic_), 7.58 (1H; C-*H*_aromatic_), 7.31 (2H; d; C-*H*_aromatic_), 7.28 (1H; d; C-*H*_aromatic_), 7.01 (1H; s; C-*H*_aromatic_) ppm. ^13^C-NMR (75 MHz, DMSO-*d*_6_) δ: 157.3, 155.9, 133.7, 131.1, 129.5, 128.9, 128.6, 125.5, 119.0, 118.1, 114.4 ppm. Analysis calculated for C_16_H_12_N_4_O_4_: C, 59.26; H, 3.73; N, 17.28. Found: C, 59.1; H, 3.69; N, 17.44. Mass calculated: *m*/*z* 324.09; Found LRMS: *m*/*z* 325.09 [M + H]^+^.

*3-((2-(4-Hydroxybenzoyl)hydrazono)methyl)-4-phenyl-1,2,5-oxadiazole 2-oxide* (**8b**): White solid. Yield: 38.5%, mp: 221–222.3 °C. IR νmax (cm^−1^; KBr pellets): 3412 (O-H), 1635 (C=O amide), 1616 (C=N), 1400 (N=O), 1294 (N-O) and 684 (C-H aromatic). ^1^H-NMR (300 MHz, DMSO-*d*_6_) δ: 10.24 (1H; O-*H*), 8.32 (1H; s; C-*H*_imine_), 7.96 (2H; d; *J* = 8 Hz; C-*H*_aromatic_), 7.79 (2H; dd; C-*H*_aromatic_), 7.60 (2H; d; *J* = 8 Hz; C-*H*_aromatic_), 7.56 (1H; C-*H*_aromatic_), 6.85 (2H; dd; C-*H*_aromatic_) ppm. ^13^C-NMR (75 MHz, DMSO-*d*_6_) δ: 166.1, 161.0, 156.7, 155.8, 151.2, 132.7, 131.3, 129.5, 128.3, 127.9, 124.0, 116.5, 118.4, 113.7 ppm. Analysis calculated for C_16_H_12_N_4_O_4_: C, 59.26; H, 3.73; N, 17.28. Found: C, 58.98; H, 3.75; N, 17.52. Mass calculated: *m*/*z* 324.09; Found LRMS: *m*/*z* 325.09 [M + H]^+^.

*5-((2-(3-Hydroxybenzoyl)hydrazono)methyl)benzo[c][1,2,5]oxadiazole 1-oxide* (**13a**): Mustard yellow solid. Yield: 50.9%, mp: 263.2–264 °C. IR νmax (cm^−1^; KBr pellets): 3431 (O-H), 1654 (C=O amide), 1616 (C=N), 1400 (N=O), 1298 (N-O), 794 (C-H aromatic) and 745 (C-H aromatic). NMR ^1^H (300 MHz, DMSO-*d*_6_) δ: 9.80 (1H; O-*H*), 8.46 (1H; s; C-*H*_imine_), 8.01 (1H; C-*H*_aromatic_), 7.88 (1H; C-*H*_aromatic_), 7.87 (1H; C-*H*_aromatic_), 7.36 (1H; d; *J* = 7.6 Hz; C-*H*_aromatic_), 7.34 (1H; dd; *J* = 7.6 Hz; C-*H*_aromatic_), 7.31 (1H; m; C-*H*_aromatic_), 7.01 (1H; dd; C-*H*_aromatic_), 4.69 (1H; N-*H*_amide_) ppm. ^13^C-NMR (75 MHz, DMSO-*d*_6_) δ: 145.0, 130.7, 129.6, 119.0, 118.6, 118.1, 114.5, 113.2 ppm. Analysis calculated for C_14_H_10_N_4_O_4_: C, 56.38; H, 3.38; N, 18.78. Found: C, 56.05; H, 3.13; N, 19.04. Mass calculated: *m*/*z* 298.07; Found LRMS: *m*/*z* 299.07 [M + H]^+^.

*5-((2-(4-Hydroxybenzoyl)hydrazono)methyl)benzo[c][1,2,5]oxadiazole 1-oxide* (**13b**): Yellow solid. Yield: 68%, mp: 295–297 °C. IR νmax (cm^−1^; KBr pellets): 3336 (O-H), 3081 (C-H aromatic), 1654 (C=O amide), 1607 (C=N), 1507 (N=O) and 795 (C-H aromatic). ^1^H-NMR (300 MHz, DMSO-*d*_6_) δ: 10.28 (1H; O-*H*), 8.45 (1H; s; C-*H*_imine_), 7.83 (2H; dd; *J* = 8.2 Hz; C-*H*_aromatic_), 6.88 (2H; dd; *J* = 8.2 Hz; C-*H*_aromatic_) ppm. ^13^C-NMR (75 MHz, DMSO-*d*_6_) δ: 164.6, 143.0, 130.3, 128.6, 124.0, 114.8 ppm. Analysis calculated for C_14_H_10_N_4_O_4_: C, 56.38; H, 3.38; N, 18.78. Found: C, 56.42; H, 3.63; N, 18.55. Mass calculated: *m*/*z* 298.07; Found LRMS: *m*/*z* 299.07 [M + H]^+^.

#### 3.2.2. General Procedures for the Preparation of Compounds (**4a**,**b**, **9a**,**b**, and **14a**,**b**)

A mixture of *N*-acylhydrazone derivatives (**3a**,**b**, **8a**,**b** and **13a**,**b**) (1.15 mmol), acetylsalicylic acid (1 mmol) (1.0 mmol), dicyclohexylcarbodiimide (1.0 mmol), dimethylaminopyridine (0.1 mmol), and 20 mL of anhydrous dichloromethane was stirred under nitrogen at 0 °C for 15 h. Subsequently the mixture was filtered and the solvent was evaporated at reduced pressure. The compounds were purified by silica gel column chromatography, using ethyl acetate:hexane (70:30) at the mobile phase to give the compounds variable yields (21.6%−39.1%).

*(E)-3-((2-(3-((2-Acetoxybenzoyl)oxy)benzoyl)hydrazono)methyl)-4-methyl-1,2,5-oxadiazole 2-oxide* (**4a**): Pale yellow solid. Yield: 23.07%, mp: 132–135.4 °C. IR νmax (cm^−1^; KBr pellets): 3431 (N-H), 1697 (C=O ester), 1643 (C=O amide), 1616 (C=N), 1462 (C-H methyl) and 1157 (C-O ester). ^1^H-NMR (300 MHz, DMSO-*d*_6_) δ: 8.49 (1H; s; C-*H*_imine_), 8.03 (1H; dd; C-*H*_aromatic_), 8.00 (1H; s; C-*H*_aromatic_), 7.93 (1H; C-*H*_aromatic_), 7.90 (1H; C-*H*_aromatic_), 7.86 (1H; t; *J* = 7.8 Hz; C-*H*_aromatic_), 7.66 (1H; d; C-*H*_aromatic_), 7.63 (1H; t; *J* = 7.8 Hz; C-*H*_aromatic_), 7.60 (1H; d; C-*H*_aromatic_), 7.05 (1H; N-*H*), 2.41 (3H; s; C-*H_3_*_furoxan_), 2.01 (3H; s; COC*H_3_*) ppm. ^13^C-NMR (75 MHz, DMSO-*d*_6_) δ: 136.0, 135.0, 130.6, 129.8, 129.7, 126.0, 125.4, 121.2, 115.0, 20.3, 8.77 ppm. Analysis calculated for C_20_H_16_N_4_O_7_: C, 56.61; H, 3.80; N, 13.20. Found: C, 56.28; H, 3.66; N, 13.57. Mass calculated: *m*/*z* 424.10; Found LRMS: *m*/*z* 425.10 [M + H]^+^.

*(E)-3-((2-(4-((2-Acetoxybenzoyl)oxy)benzoyl)hydrazono)methyl)-4-methyl-1,2,5-oxadiazole 2-oxide* (**4b**): Pale yellow solid. Yield: 26.44%, mp: 179.3–180 °C. IR νmax (cm^−1^; KBr pellets): 3441 (N-H), 1747 (C=O ester), 1639 (C=O amida), 1616 (C=N), 1458 (C-H methyl), 1168 (C-O ester). ^1^H-NMR (300 MHz, DMSO-*d*_6_) δ: 8.47 (1H; s; C-*H*_imine_), 8.20 (1H; dd; *J* = 8.1 Hz; C-*H*_aromatic_), 7.96 (1H; t; C-*H*_aromatic_), 7.81 (2H; dd; *J* = 8.1 Hz; C-*H*_aromatic_), 7.53 (1H; d; C-*H*_aromatic_), 7.36 (1H; t; C-*H*_aromatic_), 6.87 (2H; dd; C-*H*_aromatic_), 2.44 (3H; s; C-*H_3_*_furoxan_), 2.28 (3H; s; COC*H_3_*) ppm. ^13^C-NMR (75 MHz, DMSO-*d*_6_) δ: 135.0, 131.4, 129.8, 129.0, 126.7, 121.6, 115.0, 20.9, 9.0 ppm. Analysis calculated for C_20_H_16_N_4_O_7_: C, 56.61; H, 3.80; N, 13.20. Found: C, 56.87; H, 4.03; N, 13.38. Mass calculated: *m*/*z* 424.10; Found LRMS: *m*/*z* 425.10 [M + H]^+^.

*(E)-3-((2-(3-((2-Acetoxybenzoyl)oxy)benzoyl)hydrazono)methyl)-4-phenyl-1,2,5-oxadiazole 2-oxide* (**9a**): Yellow solid. Yield: 39.09%, mp: 119.1–122.7 °C. IR νmax (cm^−1^; KBr pellets): 3431 (N-H), 1743 (C=O ester), 1650 (C=O amide), 1616 (C=N), 1452 (C-H methyl), 1203 (C-O ester) and 693 (C-H aromatic). ^1^H-NMR (300 MHz, DMSO-*d*_6_) δ: 8.37 (1H; s; C-*H*_imine_), 8.20 (1H; dd; C-*H*_aromatic_), 7.97 (2H; d; *J* = 7.8 Hz; C-*H*_aromatic_), 7.88 (1H; d; C-*H*_aromatic_), 7.81 (1H; C-*H*_aromatic_), 7.80 (1H; d; C-*H*_aromatic_), 7.78 (1H; d; C-*H*_aromatic_), 7.65 (1H; d; *J* = 7.8 Hz; C-*H*_aromatic_), 7.62 (1H; t; C-*H*_aromatic_), 7.58 (2H; d; C-*H*_aromatic_), 7.52 (1H; s; C-*H*_aromatic_), 7.34 (1H; d; C-*H*_aromatic_), 2.28 (3H; s; C-*H_methyl_*) ppm. ^13^C-NMR (75 MHz, DMSO-*d*_6_) δ: 135.0, 133.0, 131.4, 131.0, 128.8, 128.5, 125.2, 125.1, 124.7, 124.0, 121.0, 120.7, 20.8 ppm. Analysis calculated for C_25_H_18_N_4_O_7_: C, 61.73; H, 3.73; N, 11.52. Found: C, 61.56; H, 3.91; N, 11.77. Mass calculated: *m*/*z* 486.12; Found LRMS: *m*/*z* 487.12 [M + H]^+^.

*(E)-3-((2-(4-((2-Acetoxybenzoyl)oxy)benzoyl)hydrazono)methyl)-4-phenyl-1,2,5-oxadiazole 2-oxide* (**9b**): Yellow solid. Yield: 38.46%, mp: 139.6–141.1 °C. IR νmax (cm^−1^; KBr pellets): 3421 (N-H), 1751 (C=O ester), 1639 (C=O amide), 1616 (C=N), 1458 (C-H methyl), 1195 (C-O ester) and 692 (C-H aromatic). ^1^H-NMR (300 MHz, DMSO-*d*_6_) δ: 8.36 (1H; s; C-*H*_imine_), 8.20 (1H; t; C-*H*_aromatic_), 7.96 (2H; d; *J* = 7.9 Hz; C-*H*_aromatic_), 7.80 (2H; dd; C-*H*_aromatic_), 7.62 (1H; dd; C-*H*_aromatic_), 7.60 (2H; d; *J* = 7.9 Hz; C-*H*_aromatic_), 7.51 (1H; s; C-*H*_aromatic_), 7.42 (1H; d; C-*H*_aromatic_), 7.35 (2H; dd; C-*H*_aromatic_), 7.32 (1H; d; C-*H*_aromatic_), 2.30 (3H; s; C-*H_methyl_*) ppm. ^13^C-NMR (75 MHz, DMSO-*d*_6_) δ: 135.1, 133.0, 131.8, 131.0, 129.0, 128.7, 126.4, 124.0, 122.0, 21.0 ppm. Analysis calculated for C_25_H_18_N_4_O_7_: C, 61.73; H, 3.73; N, 11.52. Found: C, 61.92; H, 3.91; N, 11.8. Mass calculated: *m*/*z* 486.12; Found LRMS: *m*/*z* 487.12 [M + H]^+^.

*(E)-5-((2-(3-((2-Acetoxybenzoyl)oxy)benzoyl)hydrazono)methyl)benzo[c][1,2,5]oxadiazole 1-oxide* (**14a**): Yellow solid. Yield: 21.6%, mp: 182–184.5 °C. IR νmax (cm^−1^; KBr pellets): 3414 (N-H), 1747 (C=O ester), 1635 (C=O amide), 1616 (C=N), 1400 (C-H methyl) and 1298 (N-O). ^1^H-NMR (300 MHz, DMSO-*d*_6_) δ: 8.51 (1H; s; C-*H*_imine_), 8.00 (1H; *J* = 7.7 Hz; C-*H*_aromatic_), 7.85 (1H; *J* = 7.7 Hz; C-*H*_aromatic_), 7.83 (1H; t; C-*H*_aromatic_), 7.69 (1H; t; C-*H*_aromatic_), 7.60 (1H; *J* = 7.7 Hz; C-*H*_aromatic_), 7.58 (1H; m; C-*H*_aromatic_), 7.40 (1H; t; C-*H*_aromatic_) 5.51 (1H; N-*H*), 2.31 (3H; s; C-*H_methyl_*) ppm. ^13^C-NMR (75 MHz, DMSO-*d*_6_) δ: 146.0, 131.2, 130.0, 126.1, 126.0, 125.5, 121.4, 119.6, 21.2 ppm. Analysis calculated for C_23_H_16_N_4_O_7_: C, 60.00; H, 3.50; N, 12.17. Found: C, 60.29; H, 3.19; N, 12.41. Mass calculated: *m*/*z* 460.10; Found LRMS: *m*/*z* 461.10 [M + H]^+^.

*(E)-5-((2-(4-((2-Acetoxybenzoyl)oxy)benzoyl)hydrazono)methyl)benzo[c][1,2,5]oxadiazole 1-oxide* (**14b**): Yellow solid. Yield: 22.68%, mp: 197–200 °C. IR νmax (cm^−1^; KBr pellets): 3419 (N-H), 1751 (C=O ester), 1653 (C=O amide), 1620 (C=N), 1400 (C-H methyl), 1202 (C-O ester) and 640 (C-H aromatic). ^1^H-NMR (300 MHz, DMSO-*d*_6_) δ: 8.50 (1H; s; C-*H*_imine_), 8 (1H; C-*H*_aromatic_), 7.98 (2H; dd; *J* = 8 Hz; C-*H*_aromatic_), 7.89 (1H; C-*H*_aromatic_), 7.33 (2H; dd; *J* = 8 Hz; C-*H*_aromatic_), 5.54 (1H; N-*H*), 2.32 (3H; s; C-*H_methyl_*) ppm. ^13^C-NMR (75 MHz, DMSO-*d*_6_) δ: 145.0, 131.1, 130.4, 129.9, 129.4, 121.6, 21.0 ppm. Analysis calculated for C_23_H_16_N_4_O_7_: C, 60.00; H, 3.50; N, 12.17. Found: C, 60.35; H, 3.66; N, 12.38. Mass calculated: *m*/*z* 460.10; Found LRMS: *m*/*z* 461.10 [M + H]^+^.

### 3.3. Pharmacology

#### 3.3.1. Animals

Adult male Swiss albino mice (20−35 g) and Wistar rats (200–250 g) were used in the experiments. They were housed in single-sex cages under a 12 h light:12 h dark cycle in a controlled-temperature room (22 ± 2 °C). The animals had free access to food and water. The experiments were performed after the protocol was approved by The Research Ethics Committee of the School of Pharmaceutical Sciences, UNESP, Araraquara (CEP/FCFAR/2011). All experiments were performed in accordance with the current guidelines for the care of laboratory animals and the ethical guidelines for the investigation of experimental pain in conscious animals.

#### 3.3.2. Antiplatelet Activity

Five rats (Wistar; 200–250 g) were previously anesthetized by inhalation of isoflurane using a vaporizing system. Animal breathing was monitored during all procedures. Blood was withdrawn from each rat’s aorta artery and mixed with 3.8% trisodium citrate (9:1 *v*/*v*). The animals were then euthanized by deepening of anesthesia. Platelet-rich plasma (PRP) was prepared by centrifugation at 375*× g* for 10 min at room temperature. The platelet-poor plasma (PPP) was prepared by centrifugation of the pellet at 1800*×* *g* for 10 min at room temperature. Platelet aggregation was monitored by the turbidimetric method of Born and Cross [16] using a Chrono-Log aggregometer. PRP (300 μL) was incubated at 37 ^o^C for 1 min with continuous stirring at 900 rpm. ADP (10 μM), type I collagen (5 μg/mL; molecular weight of 115–130 kDa) and arachidonic acid (100 μM) were used to induce platelet aggregation. All compounds (**3a**,**b**, **4a**,**b**, **8a**,**b**, **9a**,**b**, **13a**,**b** and **14a**,**b**) at 150 μM or vehicle DMSO (0.5% *v*/*v*) were added to the PRP samples 1 min before addition of the aggregating agent (ADP, 10 μM; collagen, μg/mL; or arachidonic acid, 100 μM). Acetylsalicylic acid (AAS, 150 μM) was used as positive control. The results were expressed as an inhibition percentage. The statistical analysis was performed using ANOVA followed by Tukey’ s test.

#### 3.3.3. *In vitro* Nitric Oxide Production

Test compounds, previously solubilized in DMSO (20 μL), were added to a 2 mL solution (50 mM phosphate buffer (pH 7.4)/methanol (1:1, *v*/*v*)) containing 5 mM l-cysteine. For all test compounds the final concentration was 10^−4^ M. This mixture was incubated for 1 h at 37 °C. After incubation, 250 μL of Griess reagent was added to 1 mL of this mixture. The absorbance was measured at 540 nm using a Shimadzu UV-2501PC spectrophotometer (Kyoto, Japan). A calibration curve of standard sodium nitrite was used to quantify the % NO_2_ in the medium [17,27]. 

#### 3.3.4. *In vivo* Antithrombotic Activity 

*In vivo* pulmonary thromboembolism was induced in mice according to previously described procedures [18,19]. Ten Swiss male mice (25−30 g) were used in each test group and the control group. Compounds (**3a**,**b**, **4a**,**b**, **8a**,**b**, **9a**,**b**, **13a**,**b** and **14a**,**b**) and acetylsalicylic acid were given orally (100 µmol/10 g, 0.1 mL/10 g) using a 2% carboxymethylcellulose suspension as the vehicle. After 60 min, an intravenous injection of the thrombogenic stimulus containing a mixture of collagen (11 mg/kg) plus epinephrine (0.7 mg/kg) was administered through the tail vein. The dose of the pro-thrombotic mixture was previously characterized using a dose/response curve able to reliably reproduce 80%–90% mortality rate or animal paralysis [15]. The loss of the righting reflex was considered an indication of animal’s paralysis in the control group. The rates of animal survival and paralysis were evaluated 15 min after the administration of the thrombogenic stimulus. After this time, the animals were euthanized using carbon dioxide. In the control group, there were five deaths. For ASA and **3a**, **3b**, **4a**, **8a**, **8b***,* and **9a**, there were two deaths per group. The protection against acute pulmonary thromboembolism (percentage) was calculated as follows: [1 − (paralyzed + death animals)/total animals] × 100. The chi-square test was used to examine the difference between the control and treated groups.

#### 3.3.5. *In Silico* Studies

Molecular modeling studies were performed with Maestro software Maestro v9.1, software available from Schrödinger Inc, New York, NY, USA [28]. The ligand-bound crystallographic structure of cyclooxygenase (COX-1) is available in the Protein Data Bank. In this study, celecoxib crystalized in the active site of COX-1 (pdb code: 3KK6) was selected for docking studies [29]. The crystallographic water molecules, ions and cofactors were deleted, hydrogen atoms were added, and formal charges along with bond orders were assigned to the structure. The ligand (**14a**) was prepared using LigPrep (Schrödinger) [30]. Using OPLS 2005 force field, 32 stereochemical structures per ligand were generated. The grid was generated as a cubic box of 10 Å × 10 Å × 10 Å centered on the active site residues. The SP flexible ligand docking was performed using Glide within Schrödinger-Maestro v9.1 [31]. The final score was obtained for energy-minimized poses and the best-docked pose with lowest glide score was selected for **14a**. *In silico* physico-chemistry and pharmacokinetic properties were determined using the pkCSM database [22].

#### 3.3.6. *In Vivo* Genotoxicity Study 

Compound **14a** (250 mg/kg) and negative control (1% carboxymethylcellulose (CMC) suspension and 0.2% tween; 0.3 mL) were administered to the animals orally. The positive control group received cyclophosphamide (50 mg/kg; i.p.). Groups of 10 Swiss mice of both sexes (25−30 g) were used in each test and control group. After 30 h, the animals were anesthetized using a mixture of ketamine/xylazine (100/10 mg/kg; i.p*.*), and 0.5 mL of blood were collected from the tail vein and added to laminas pre-stained with acridine orange. The animals were euthanized via deepening of anesthesia. One thousand reticulocytes per animal were counted, and the frequency of micronucleated cells was registered. These results were tested with analysis of variance (ANOVA). When *p* < 0.05, the average values for the treatments were compared using the Tukey method, calculating the minimum significant difference at α = 0.05.

## 4. Conclusions

In this study, we synthesized and characterized a series of furoxan and benzofuroxan derivatives (**3a**,**b**, **4a**,**b**, **8a**,**b**, **9a**,**b**, **13a**,**b**, and **14a**,**b)**. All compounds demonstrated antiplatelet activity induced by ADP, collagen and arachidonic acid. The furoxan derivatives (**3a**,**b**, **4a**,**b**, **8a**,**b**, and **9a**,**b**) released NO *in vitro;* however, this effect was not observed for benzofuroxan derivatives (**13a**,**b** and **14a**,**b**). The presence of *N*-oxide in benzofuroxan derivatives seems to be relevant for hydrogen bond interactions with His90 and Ser516 in the active site of COX-1. Furthermore, benzofuroxan derivatives (**13a**,**b** and **14a**,**b**) were the most effective compounds in the pulmonary thromboembolism model induced by collagen and epinephrine, providing a survival rate up to 80%. Finally, the micronucleus test using peripheral blood cells of mice showed that the benzofuroxan derivative **14a** was non-genotoxic. Based on these results, **14a** has emerged as a new prototype useful for the prevention of atherothrombotic events. 
